# An abnormal secretion of soluble mediators contributes to the hematopoietic-niche dysfunction in low-risk myelodysplastic syndrome

**DOI:** 10.1038/bcj.2015.97

**Published:** 2015-11-27

**Authors:** M Martini, S Capodimonti, M G Iachininoto, A Cocomazzi, E R Nuzzolo, M T Voso, L Teofili, L M Larocca

**Affiliations:** 1 Istituto di Anatomia Patologica, Università Cattolica del Sacro Cuore, Rome, Italy; 2 Istituto di Ematologia, Università Cattolica del Sacro Cuore, Rome, Italy; 3 Department of Biomedicine and Prevention, Università di Roma Tor Vergata, Roma, Italy

The bone marrow (BM) niche has a pivotal role in human hematopoiesis, both promoting hematopoietic stem cells (HSC) proliferation and differentiation and favoring the HSC maintenance, particularly after an injury.^1, 2^ Although the HSC niche remains incompletely defined and the distinction between endosteal and vascular niches is outdated, recent progresses have been made in elucidating location and cellular composition of BM niches: the niche is perivascular, located in the trabecular bone and mainly consists of HSC, endothelial and mesenchymal stromal cells.^1, 2^

Emerging evidences indicate that BM niche dysfunction may be involved in the pathogenesis of several hematologic diseases such as Philadelphia (Ph)-negative myeloproliferative neoplasms and myelodysplastic syndromes (MDS), pointing the attention on a BM microenvironment alteration, as well as on the genetic and epigenetic defects of the hematopoietic cells.^2, 3, 4^ In this way, we demonstrated that molecular alterations occurring in negative myeloproliferative neoplasms, such as *SOCS* gene hypermethylation and *JAK2*^*V617F*^ mutation, are also detected in the endothelial cell compartment.^5^ Moreover, we recently carried out an *in vitro* elegant and simple model of the HSC niche using normal CD34+ totipotent cells and expanded endothelial colony forming cells (ECFCs), isolated from the peripheral blood of patients with low-risk MDS or healthy donors, as a surrogate of an important component of the HSC niche, the endothelial cells.^6^ Inducing the three-lineage differentiation, we demonstrated an impairment of talking between endothelial and hematopoietic cells that affected the hematopoietic differentiation in comparison with control counterparts. Molecular characterization of MDS ECFCs revealed that these cells frequently have a hypermethylated phenotype, an altered gene expression profile (including adhesion, the Wingless and int-Wnt pathway, angiogenesis, cell activation, cell survival and apoptosis pathways) and miRNA expression profile.^6^

Soluble mediators (growth factors, cytokines and chemokines), secreted in the BM microenvironment by accessory cells (such as fibroblasts, T lymphocytes and endothelial cells) and CD34+ cells, have a central role in the intercellular cross-talk network and/or autocrine/paracrine regulatory loops that supervise normal hematopoiesis.^1, 2, 7^ Although the composition, the biological functions and the fine molecular regulation of this network is not clear,^7^ several studies demonstrated that it is often deregulated in different hematopoietic diseases even with a prognostic value.^4, 8^

The aim of this study is to assess the pattern of soluble mediators in our *in vitro* model of HSC niche, in order to evaluate if eventual imbalances might underlie the ineffective hematopoiesis of myelodysplasia.

Blood samples were obtained from 10 patients (5 males and 5 females, median age 63 years, range 46–74) affected by low-risk MDS according to the International Prognostic Scoring System.^6^ At the time of study, patients were not receiving antiproliferative or demethylating drugs. Five healthy blood donors (3 males and 2 females, median age 61 years, range 52–76) were used as controls. All blood samples were obtained after informed consent. The study was approved by the institutional Ethic Committee of Catholic University of Rome.

ECFCs were obtained from peripheral blood of MDS patients or from healthy blood donors according to the method previously described.^5, 6^ The soluble mediator concentration was evaluated by incubating, normal cord blood CD34+ cells over ECFC layers, obtained from healthy donors and low-risk MDS patients.^6^ The CD34+ cells were plated in direct contact with endothelial cells layers in 24 multi-well plates at 2 × 10^5^ cells/ml concentration. Supernatants of the co-cultures were collected at baseline and after 24 and 48 h, stored at −80 °C until soluble mediator dosages.

Multiplex analysis of cytokines, chemokines and growth factors in supernatants of the co-cultures was performed by using Bio-Plex Pro Human Cytokine 27-Plex Immunoassay (Bio-Rad Laboratories, Hercules, CA, USA), according to the manufacturer's manual and Bio-Plex Manager MP Software Version 1.0 (Bio-Rad Laboratories).^8^ In particular, we measured interleukin (IL)-1β, IL-1ra, IL-2, IL-4, IL-5, IL-6, IL-7, IL-8, IL-9, IL-10, IL-12 (p70), IL-13, IL-15, IL-17, basic fibroblast growth factor (basic), eotaxin, granulocyte colony-stimulating factor, granulocyte macrophage colony-stimulating factor, interferon-gamma (IFN-γ), interferon-gamma-induced protein 10 (IP-10), monocyte chemoattractant protein (MCP-1), macrophage inflammatory protein (MIP)-1α, MIP-1β, platelet-derived growth factor-bb, regulated and normal T-cell expressed and secreted, tumor necrosis factor (TNF)-α and endothelial growth factor (VEGF). The assay was performed in duplicate and the concentration values (in pictograms per milliliter) were interpolated from standard curves.

Statistical comparison of continuous variables was performed with the Unpaired *t*-test, as appropriate (GraphPad-Prism 5 software, San Diego, CA, USA). Comparison of categorical variables was performed using chi-square statistic and the Fisher's exact test. *P-*values <0.05 were considered as statistically significant.

When we compared the expression of the different cytokines, growth factors and chemokines between MDS and healthy co-cultures, we found a different and significant expression of several soluble mediators (Table 1). In addition, despite the different levels at 24 and 48 h of co-cultures, each mediator showed a trend and a significant difference in both determinations (data not shown). In particular, a group of mediators showed a higher and significant level in MDS co-cultures in comparison with the normal counterpart: among them, IL-1β, IL-8, IFN-γ, TNF-α, platelet-derived growth factor-bb and VEGF had a significant overexpression (*P<*0.001; Figure 1, panel a). Moreover, also the levels of regulated and normal T-cell expressed and secreted, granulocyte colony-stimulating factor, granulocyte macrophage colony-stimulating factor and IP-10 were significantly higher (*P<*0.01). In contrast, the expression of IL-5, IL-15, IL-17, IL-9, MIP-1β and IL-2 were only slightly increased.

Conversely, we found that several mediators were under-expressed in MDS as compared with control (Table 1). In particular, IL-4, IL-10, IL-13 and IL-1ra had a significant lower level (Table 1 and Figure 1, panel b), Eotaxin with a borderline *P*-value, while MIP-1α, fibroblast growth factor, MCP-1, IL-6, IL-7 and IL-12 did not have a significant downregulation.

We recently demonstrated in our *in vitro* model of hematopoietic niche that healthy endothelial progenitor cells supported the expansion and differentiation of cord blood CD34+ placed in a direct contact with ECFC. On the contrary, ECFC from patients with low-risk MDS have several molecular alterations and fail in adequately sustaining hematopoiesis.^6^ In this work we found that MDS endothelial progenitor cells produce an abnormal pattern of cytokines, chemokines and growth factors, suggesting that the soluble mediator network could be deeply perturbed in the MDS niche.

Comparing the expression of soluble mediators between the MDS and normal ECFC co-cultures, we observed two distinct mediator subgroups over or under produced, respectively. Among molecules detected at higher levels in MDS than in controls, IL-1β, IL-8, IFN-γ, regulated and normal T-cell expressed and secreted, and TNF-α exert an acknowledged pro-inflammatory and chemo- attractant effect in several diseases,^8, 9^ while VEGF and platelet-derived growth factor-bb mainly regulate endothelial cell proliferation and angiogenesis.^10^ Furthermore, granulocyte colony-stimulating factor and granulocyte macrophage colony-stimulating factor are key factors for hematopoietic cell differentiation, as well as important inflammation mediators.^9, 11^ On the opposite, several other cytokines detected at lower levels in MDS than in normal co-cultures, such as IL-4, IL-10 and IL-13, mainly elicit anti-inflammatory effects.^8, 9^

A compelling body of evidences demonstrated that an overproduction of pro-inflammatory cytokines such as IFN-γ, TNF-α, IL-1β and granulocyte macrophage colony-stimulating factor are implicated in hematopoietic failure occurring in chronic inflammatory diseases as well as in MDS.^3, 4, 8, 9, 12^ The high expression of VEGF in our MDS co-culture model agrees with the evidence of an increased BM microvessel density and with a poor prognosis in MDS patients with high VEGF plasma levels.^13^ In addition, several pro-inflammatory cytokines, such as TNF-α and IL-1β are able to induce the VEGF production.^9^ As a whole, our results are in partial concordance with those of Kornblau *et al.,*^8^ demonstrating abnormal levels of various cytokines and chemokines in serum of patients with leukemia and MDS. This partial concordance could be due by the different types of sample: the peripheral serum with respect to supernatant of our *in vitro* model, consisting in the interaction of only two cell types.^6, 8^

We have recently described the increased expression of adhesion molecules such as ICAM-1,VCAM-1, vWF and SELL in MDS ECFC.^6^ Interestingly, many cytokines and, in particular, those with pro-inflammatory effect (IFN-γ and TNF-α, for example) are able to directly or indirectly induce the expression of several adhesion molecules, especially in endothelial cells.^14^ Similarly, both anti-inflammatory and pro-inflammatory cytokines are able to modulate the Wnt pathway,^15^ that we found implicated in the impaired hematopoietic differentiation induced by MDS endothelial cells.^8^

Although the major part of the soluble mediators has a pleiotropic, bidirectional and synergic effect both in normal and pathologic processes, our results seems to increasingly support the novel concept of a myelodysplatic niche disease in which the pathological process rather affects the entire BM microenvironment. Therefore, the altered cytokines and chemokines pathway could have a centrall role in the MDS niche dysfunction, modifying regenerative capacity and the differentiation ability of the HSC.^3, 4, 6^

In conclusion, our observations add a new tile to the complex mosaic of the MDS pathogenesis, highlighting the possible role of altered soluble mediators, particularly of those with a pro-inflammatory and angiogenetic effects, reinforcing the idea of a primary dysfunctions of the HSC niche as important drivers for myelodysplasia and then offering an innovative perspective for a new and different therapeutic approach.

## Figures and Tables

**Figure 1:**
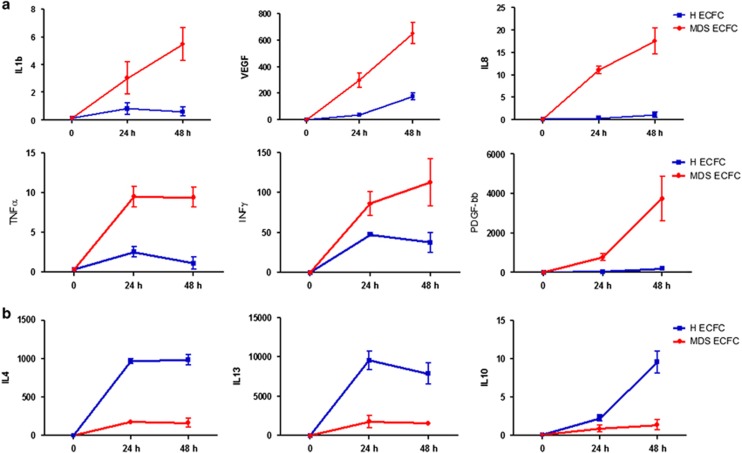
(**a**) Shows the significant upregulated expression of IL-1β, VEGF, IL-8, IFN-γ, TNF-α and platelet-derived growth factor-bb in MDS co-cultures (red line) with respect to the healthy counterpart (blue line). Conversely, (**b**) shows the significant downregulated expression of IL-4, IL-10 and IL-13 in MDS co-cultures (red line) with respect to the healthy counterpart (blue line).

**Table 1:** Comparison of the expression of soluble mediators in co-cultures of CD34+ and healthy ECFC and co-cultures of CD34+ and MDS ECFC (analysis after 48 h). The *P*-value indicates the statistical significance

*Soluble mediator*	*Fold 48h MDS* vs *H*	P-*value*
IL-1β	9.15	<0.001
IL-8	6.72	<0.001
IFN-γ	3.01	<0.001
TNF-α	8.40	<0.001
PDGF-bb	18.53	<0.001
VEGF	3.73	<0.001
RANTES	1.23	<0.01
G-CSF	2.14	<0.01
GM-CSF	5.32	<0.01
IP-10	4.03	<0.01
IL-5	0.03	NS
IL-15	0.03	NS
IL-17	1.15	NS
IL-9	0.07	NS
MIP-1β	0.89	NS
IL-2	0.23	NS
IL-4	−6.02	<0.001
IL-10	−6.99	<0.001
IL-13	−5.15	<0.001
IL-1ra	−2.38	<0.01
Eotaxin	−0.98	<0.05
MIP-1α	−0.07	NS
FGF	−0.74	NS
MCP-1	−1.46	NS
IL-6	−0.56	NS
IL-7	−0.37	NS
IL-12 (p70)	−0.26	NS

Abbreviations: FGF, fibroblast growth factor; G-CSF, granulocyte colony-stimulating factor; GM-CSF, granulocyte macrophage colony-stimulating factor; IFN-γ, interferon-gamma; IP-10, interferon-gamma-induced protein 10; MCP, monocyte chemoattractant protein; MIP, macrophage inflammatory protein; NS, not significant; PDGF, platelet-derived growth factor; RANTES, regulated and normal T-cell expressed and secreted; TNF, tumor necrosis factor; VEGF, endothelial growth factor.
